# Personality traits as mediators in the association between *SIRT1* rs12415800 polymorphism and depressive symptoms among Chinese college students

**DOI:** 10.3389/fpsyt.2023.1104664

**Published:** 2023-04-14

**Authors:** Chenliu Wang, Lei Ji, Decheng Ren, Fan Yuan, Liangjie Liu, Yan Bi, Zhenming Guo, Fengping Yang, Yifeng Xu, Shunying Yu, Zhenghui Yi, Lin He, Chuanxin Liu, Guang He, Tao Yu

**Affiliations:** ^1^Key Laboratory for the Genetics of Developmental and Neuropsychiatric Disorders, Bio-X Institutes, Shanghai Jiao Tong University, Shanghai, China; ^2^Shanghai Key Laboratory of Psychotic Disorders, and Brain Science and Technology Research Center, Shanghai Jiao Tong University, Shanghai, China; ^3^School of Mental Health, Jining Medical University, Jining, Shandong, China

**Keywords:** depressive symptoms, personality traits, mediation analysis, neuroticism, SIRT1 rs12415800

## Abstract

**Background:**

Previous research has linked polymorphisms in the *SIRT1* gene to depressive symptoms, particularly in Chinese individuals. However, it is not clear how personality traits may contribute to this association.

**Methods:**

To explore the potential mediating effect of personality traits, we utilized a mediation model to examine the relationship between the *SIRT1* rs12415800 polymorphism and depressive symptoms in 787 Chinese college students. Depressive symptoms were assessed using the Center for Epidemiologic Studies Depression (CES-D) scale, while personality traits were measured using the Big Five Inventory (BFI).

**Results:**

Our analysis indicated a significant association between the *SIRT1* rs12415800 polymorphism and depressive symptoms, with this relationship partially mediated by the personality traits of neuroticism and conscientiousness. Specifically, individuals who were heterozygous for the rs12415800 polymorphism and had higher levels of conscientiousness were less likely to experience depressive symptoms. Conversely, those who were homozygous for the rs12415800 polymorphism and had higher levels of neuroticism were more likely to experience depressive symptoms.

**Conclusion:**

Our results suggest that personality traits, particularly neuroticism and conscientiousness, may play a critical role in the association between the *SIRT1* rs12415800 polymorphism and depressive symptoms among Chinese college students. These findings highlight the importance of considering both genetic factors and personality traits when exploring the etiology of depressive symptoms in this population.

## 1. Introduction

Depressive symptoms are a major concern among college students, with even mild symptoms having the potential to develop into clinical depression (1) and adversely affecting various aspects of their lives, such as social functioning, educational attainment, and physical and mental health (2). The prevalence of depressive symptoms has been increasing among college students worldwide, with rates ranging from 24 to 56.8% depending on the study (3–5). College life is a critical transition period associated with a range of challenges, including academic stress, environmental change, and social problems, which may contribute to the development of depression (6, 7). In addition, depressive symptoms increase the risk of suicidal ideation or behavior, making it a critical public mental health problem (8). Thus, investigation into the underlying causes of depressive symptoms can significantly enhance our understanding of this illness and aid in improving the mental health of college students.

Research indicates that depression has a moderate genetic contribution, with an estimated heritability of around 30 to 40% (9, 10). In particular, rs12415800, a gene polymorphism in the *SIRT1* gene, has been identified as the first reported genetic variant associated with major depression in the Chinese population through large-scale genome-wide association studies (11). Further investigations have confirmed the significant association of rs12415800 with major depression, revealing that individuals carrying the MDD risk allele of rs12415800 exhibit lower SIRT1 mRNA levels in Chinese postmortem brain and peripheral blood samples and display abnormal gray matter volume in the cerebellar lobe compared with those carrying the non-risk allele (12).

A recent study has also shown that the risk allele of rs12415800 might be associated with gray matter volume deficits in frontal and precuneus regions in Chinese patients with first-episode medication-naïve major depression (13). However, the link between rs12415800 variation and depression has not been consistently replicated in genome-wide association studies with large European sample sizes, suggesting that rs12415800 may be an ethnicity-specific risk factor for depression (14–16). In light of this, this study aims to investigate the correlation between the rs12415800 variant and depressive symptoms in a Chinese college population.

Several personality traits have been identified as being linked to depressive symptoms in adolescents. In particular, neuroticism has been consistently found to be positively associated with depressive symptoms in multiple studies (17–19). Conversely, personality traits such as extraversion, openness, agreeableness, and conscientiousness have been negatively associated with depressive symptoms (17, 18).

Moreover, personality traits have been identified as a mediator of the relationship between depression and heredity. Specifically, one study has shown that personality traits share a genetic basis with depressive symptoms and subjective well-being, making them an important predictor of both (20). In addition, another study found that genetic variances of two personality traits explained all subsequent genetic variances of depression and anxiety (21). These findings suggest that personality traits mediate the relationship between depression and heredity through shared genetic factors.

Our study aims to investigate the potential association between depressive symptoms and the *SIRT1* SNP rs12415800, mediated by personality traits in Chinese college students. We hypothesize that this SNP is related to depressive symptoms in this population, and that this relationship is influenced by personality traits. As previous studies have yielded inconsistent findings regarding the effects of rs12415800 in different populations, our research could provide valuable insight into the genetic underpinnings of the relationship between personality traits and depressive symptoms among Chinese college students.

## 2. Materials and methods

### 2.1. Participants

A total of 787 Chinese college students were recruited from Jining Medical University and Shanghai Jiao Tong University, representing 25 different provinces throughout China. Supplementary Material T1 provides the proportion of college students from each province. Data were collected through questionnaires, including demographic information, personality traits, and psychological health status such as depressive symptoms, anxiety, and sleep status. Informed consent was obtained from all participants prior to data collection. This study was approved by the Ethics Committee of both Jining Medical University and the Shanghai Human Genetic Resources Ethics Committee.

### 2.2. Measurements

The primary questionnaires utilized in this study were the Center for Epidemiological Survey Depression scale (CES-D) and the Big Five Inventory (BFI). The CES-D is a widely used tool for measuring depressive symptoms and has been validated for use in both general and clinical populations (22). Participants are asked to rate the frequency of depressive moods and behaviors on a four-point scale from ‘0’ (never or few) to ‘3’ (usually). Scores range from 0 to 60, with a score of 16 or higher indicating the presence of possible depressive symptoms (23). The BFI, on the other hand, was used to measure the big five personality traits: extraversion, agreeableness, conscientiousness, neuroticism, and openness. The BFI consists of 44 items, each rated on a scale from “1” (strongly disagree) to ‘5’ (strongly agree). The BFI is a widely used tool for measuring personality traits and has been validated for use among Chinese college students (8).

### 2.3. SNP selection and genotyping

The rs12415800 variant in the *SIRT1* gene was genotyped. It is located upstream of the 5′ end of the *SIRT1* gene and is the first reported gene polymorphism associated with major depression in the Chinese population through a large-sample GWAS study [CONVERGE consortium (24)]. The gene extraction and genotyping method used in this study is consistent with our previously published paper (25). In brief, DNA was extracted from peripheral venous blood using the Trizol protocol, and genotyping was performed using matrix-assisted laser desorption/ionization time of flight (MALDI-TOF) mass spectrometry on the MassARRAY Analyzer 4 platform (Sequenom, San Diego, CA, United States). Probes and primers were designed using the My-Sequenom online software Assay Design Suite v2.0.

### 2.4. Statistical analysis

The R software version 4.1.0 (R Foundation for Statistical Computing, Vienna, Austria) was used for all statistical analyzes. Quantitative data that conformed to a normal distribution were expressed as means ± standard deviation (SD) and were analyzed using *t*-tests or analysis of variance (ANOVA). Categorical data were described as frequency and percentage, and analyzed using Chi-square tests. The association between rs12415800 and depressive symptoms was analyzed using the “SNPassoc” R package in five genetic models (codominant, dominant, recessive, overdominant, and log-additive model). Mediation analyzes were conducted using the “lavaan” R package to estimate the effects of rs12415800 on depressive symptoms *via* personality, while controlling for age and gender. Model fit was assessed using Chi-square values, root mean square error of approximation (RMSEA), comparative fit index (CFI), and Tucker–Lewis index (TLI). Significance of mediation effects was determined using 1,000 bias-corrected and accelerated bootstrapping analyzes for 95% confidence intervals. RMSEA≤0.08 and CFI/TLI ≥ 0.90 were considered acceptable fit measures (26, 27).

## 3. Results

### 3.1. Descriptive statistics and correlations between main study variables

The demographic information of the study participants is presented in Table 1. Participants were classified into two groups based on their scores of depressive symptoms: those who scored higher than 16 were considered to have depressive symptoms. Our analysis revealed significant differences between the two groups in sleep status, exercise frequency, and parental temperament and personality traits. Specifically, we found that students with higher scores of depressive symptoms had higher neuroticism scores, but lower scores in extraversion, conscientiousness, agreeableness, and openness traits.

**Table 1:** Demographic characteristics of participants.

	DSs group (*n* = 348)		Control group (*n* = 439)		
	Mean	SD	Mean	SD	*p* value
Weight	61.84	16.25	60.01	14.09	0.09
Height	168.58	9.08	168.72	8.67	0.832
Personality					
Extraversion	22.81	4.3	28.24	4.99	<0.001
Agreeableness	29.56	4.21	35.43	4.9	<0.001
Conscientiousness	26.9	3.73	31.46	5.42	<0.001
Openness	31.65	4.55	35.86	5.11	<0.001
Neuroticism	25.73	3.61	20.5	4.83	<0.001
	*n*	%	*n*	%	
Residence = Urban	139	39.9	166	37.8	0.592
Child = Single	135	38.8	169	38.5	0.991
Minority = Other	9	2.6	16	3.6	0.525
Sleep					<0.001
Bad	43	12.4	14	3.2	
General	191	54.9	143	32.6	
Good	78	22.4	183	41.7	
Very bad	17	4.9	2	0.5	
Very good	19	5.5	97	22.1	
Exercise(h)					<0.001
<0.5	180	51.7	146	33.3	
>2.0	5	1.4	13	3	
0.5–1.0	129	37.1	225	51.3	
1.0–2.0	34	9.8	55	12.5	
Father. education> = High school	164	47.1	228	51.9	0.205
Mother. education> = High school	132	37.9	191	43.5	0.132
Father. character					<0.001
Cheerful	97	27.9	160	36.4	
Mild	100	28.7	160	36.4	
Rough	14	4	5	1.1	
Serious	85	24.4	79	18	
Silent	52	14.9	35	8	
Mother. character					0.001
Cheerful	132	37.9	190	43.3	
Mild	163	46.8	222	50.6	
Rough	10	2.9	3	0.7	
Serious	35	10.1	22	5	
Silent	8	2.3	2	0.5	

Furthermore, we investigated the minor allele frequency (MAF) of rs12415800, which was found to be 0.44 in our sample. Importantly, we conducted a *χ*^2^ test and found that the rs12415800 SNP was in agreement with the Hardy–Weinberg equilibrium (*p* value = 0.878).

### 3.2. Association analysis

#### 3.2.1. Association analysis of The rs12415800 polymorphism and depressive symptoms

We investigated the association between the rs12415800 polymorphism and depressive symptoms using different genetic models. Results presented in Table 2 revealed that the rs12415800 polymorphism was significantly associated with depressive symptoms under both the codominant (*p* = 0.0437) and overdominant (*p* = 0.0147) models. The rs12415800 polymorphism was also significant in codominant model (*p* < 0.05) but failed to fitting the 95% confidence interval constraint (contains zero). In particular, individuals with the heterozygous (A/G) genotype had lower levels of depressive symptoms compared to those with homozygous (G/G, A/A) genotypes, as observed in the overdominant model.

**Table 2:** Association between rs12415800 and depressive symptoms.

rs12415800	N	Mean	SE	Β(95%CI)	*p*-value
Codominant					
G/G	214	17	0.78		0.0437*
A/G	348	15.27	0.58	(−3.64, 0.1)	
A/A	137	17.73	0.95	(−1.69, 3.03)	
Dominant					
G/G	214	17	0.78		0.2332
A/G-A/A	485	15.96	0.49	(−2.86, 0.69)	
Recessive					
G/G-A/G	562	15.93	0.46		0.0916
A/A	137	17.73	0.95	(−0.28, 3.83)	
Overdominant					
G/G-A/A	351	17.28	0.6		0.0147*
A/G	348	15.27	0.58	(−3.66, −0.4)	
log-Additive					
0,1,2				(−1.07, 1.27)	0.863

Significant *P* (<0.05) values are in bold. 95%CI: 95% Confidence Interval. *p < 0.05.

Furthermore, we also explored the association between the rs12415800 polymorphism and personality traits. Results indicated that the heterozygous (AG) genotype was significantly associated with higher conscientiousness (*p* = 0.033) and lower neuroticism (*p* = 0.001) compared to the homozygous genotype under the overdominant model. These findings suggest that personality traits may contribute to the association between the rs12415800 polymorphism and depressive symptoms.

#### 3.2.2. Mediation analysis

**Figure 1:**
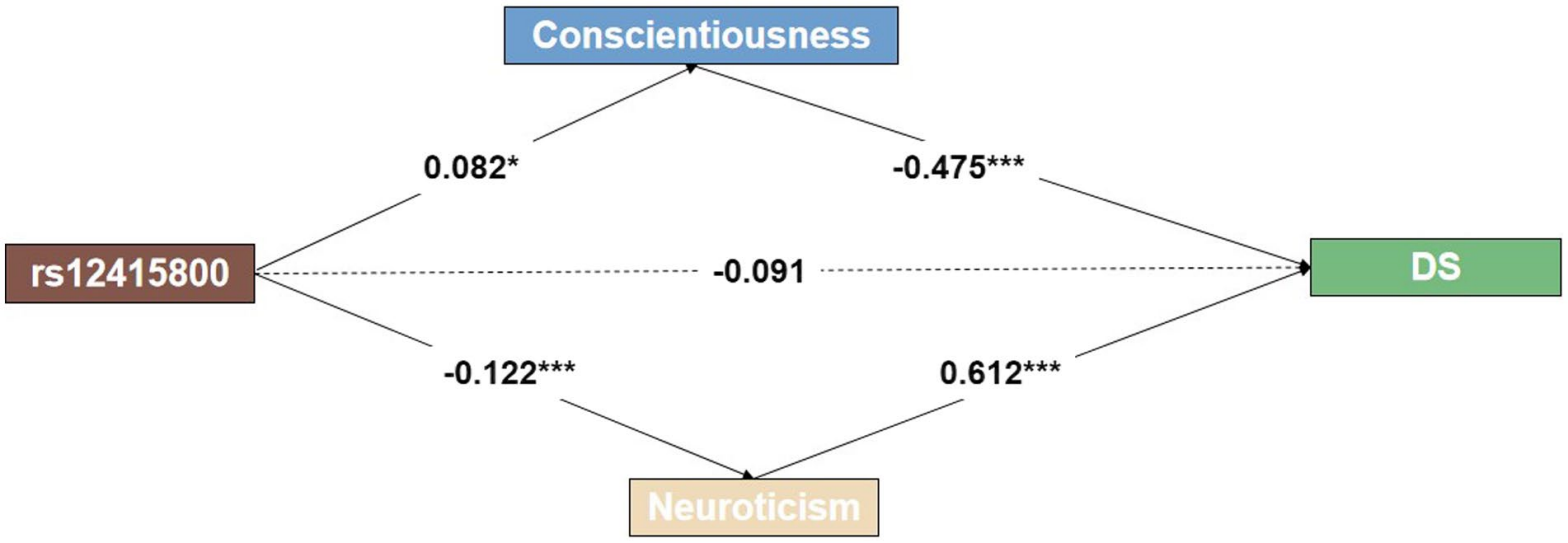
Multi-mediation plot shows an indirect effect between rs12415800, personality traits, and depressive symptoms. The dashed lines represent insignificant estimates. *p < 0.05; **p < 0.01; ***p < 0.001.

We examined the mediating role of personality traits in the relationship between the rs12415800 polymorphism and depressive symptoms while controlling for gender and age as covariates. Figure 1 shows that the rs12415800 polymorphism had a significant total effect on depressive symptoms (*p* < 0.05). However, after accounting for the effects of personality traits, there was no direct effect of the rs12415800 polymorphism on depressive symptoms. The results revealed a significant indirect effect, suggesting that the rs12415800 polymorphism partly affects depressive symptoms through the mediating role of personality traits such as conscientiousness (*β* = 0.082, *p* < 0.05) and neuroticism (*β* = −0.122, *p* < 0.001). Specifically, the study found that individuals with the heterozygous (A/G) genotype of the *SIRT1* rs12415800 polymorphism may be less likely to experience depressive symptoms by increasing their levels of conscientiousness or decreasing their levels of neuroticism.

Structural models were used to test the mediating role of personality traits in the relationship between *SIRT1* rs12415800 polymorphism and depressive symptoms while controlling for gender and age as covariates. The final model showed a good model fit: *χ*^2^(3) = 243.937, RMSEA = 0.001, CFI = 0.999, TLI = 0.999, with bootstrapping the mediating effect 1,000 times. In addition, we have found that the total indirect effect of conscientiousness and neuroticism accounts for 30.0 and 45.1%, respectively, of the overall effect in the correlation between *SIRT1* rs12415800 genotype and DS.

## 4. Discussion

This study investigated the association between rs12415800 polymorphism and depressive symptoms, as well as its relationship with personality traits. The results of the analysis indicated that individuals with the heterozygous genotype had significantly lower levels of depressive symptoms and higher levels of conscientiousness, while also exhibiting lower levels of neuroticism. Additionally, the researchers conducted a mediation analysis to explore the role of personality traits in the relationship between the rs12415800 polymorphism and depressive symptoms. The findings revealed that rs12415800 polymorphism partly affects depressive symptoms through the mediating role of personality traits. Specifically, individuals with the heterozygous genotype may be less likely to experience depressive symptoms by increasing their levels of conscientiousness or decreasing their levels of neuroticism.

Our study identified a polymorphism, rs12415800, located upstream of the 5′ end of the *SIRT1* gene, which is linked to depression and neuroticism. This polymorphism has also been reported in previous studies (CONVERGE consortium (12, 13, 24)). In a large-scale genome-wide association study (GWAS) of 5,303 Han Chinese women with recurrent and severe major depressive disorder (MDD) and 5,337 healthy female controls, two single-nucleotide polymorphisms (SNPs), rs12415800 in *SIRT1* and rs35936514 in *LHPP*, were significantly associated with MDD (CONVERGE consortium (24)). However, only rs12415800 was replicated in a separate cohort of 3,231 cases with recurrent MDD and 3,186 controls in Chinese population (12). Carriers of the MDD risk A allele of rs12415800 displayed lower SIRT1 mRNA levels in Chinese postmortem brain and peripheral blood samples compared to non-risk allele carriers, and also showed abnormal gray matter volume in the cerebellar lobe and frontal superior and precuneus regions (12, 13). It is noteworthy that subsequent Genome-Wide Association Studies (GWAS) involving substantial European samples were unable to confirm the association between rs12415800 variation and depression (14–16). Additionally, rs12415800 displays varying gene frequencies among distinct populations (24). This implies that rs12415800 might constitute an ethnically distinct vulnerability factor for depression, unique to the Chinese population.

Our study found that the *SIRT1* gene may confer protection against depressive symptoms in humans. Individuals in our samples with the heterozygous genotype for *SIRT1* at rs12415800 displayed significantly reduced levels of depressive symptoms. This protective effect may be attributed to heterozygote advantage (28), a phenomenon in genetics where individuals carrying two different alleles for a particular gene have an advantage over those carrying two copies of the same allele. For instance, *MAD1L1* (rs1107592) and *TSNARE* (rs4976976) heterozygotes showed a decreased risk of schizophrenia (29). Furthermore, variants of genes like *SLC6A4* and *ANK3* have also been associated with a higher risk of depression or bipolar disorder when present in two copies, but not when present in only one copy (30, 31), suggesting that heterozygosity for such variants may confer protection against depression.

Our study has shown that the *SIRT1* gene could potentially offer protection against depressive symptoms in humans. Notably, individuals with the heterozygous genotype for *SIRT1* at rs12415800 in our samples had significantly lower levels of depressive symptoms. This protective effect can be attributed to the heterozygote advantage, which is a genetic phenomenon where individuals carrying two different alleles for a particular gene have an advantage over those carrying two copies of the same allele (28, 32). This advantage has been observed in other genes as well, such as *MAD1L1* (rs1107592) and *TSNARE* (rs4976976) heterozygotes who have a decreased risk of schizophrenia (29), and Ank3+/− heterozygous mice which exhibited similar behavioral alterations of reduced anxiety and increased motivation for reward when compared to wild-type Ank3+/+ mice (33). While the concept of heterozygote advantage is still an active area of research and debate in genetics, and its role in mental disorders is not yet fully established, our findings suggest that the *SIRT1* gene may play a protective role in depressive symptoms.

Animal studies have shown that Sirt1 signaling has a protective effect in depressive-like animals, but dysregulation of this signaling pathway can have a critical role in depressive-like behaviors. Chronic stress decreases Sirt1 activity in the dentate gyrus, leading to elevated depression-like behaviors. However, activation of Sirt1 can block these behaviors and abnormal dendritic structures induced by chronic stress (34). The possible mechanisms of Sirt1 in depression include neurogenesis, glial activation, and neuroinflammation (35, 36). Resveratrol-triggered Sirt1 activation can reverse lipopolysaccharide (LPS)-induced depressive-like behaviors by increasing hippocampal neurogenesis and attenuating LPS-dependent overactivation of microglia in the dentate gyrus-subgranular zone (DG-SGZ) (35). Sirt1 has a neuroprotective effect in neurodegenerative diseases and can regulate downstream transcription factors such as nuclear factor erythroid 2-related factor 2 (Nrf2) and NF-κB to prevent inflammation damage, which is an important factor in the pathogenesis of depression (36, 37).

Our research has identified personality traits as a mediator for the relationship between depressive symptoms and the *SIRT1* rs12415800 variant. The correlations between personality traits and depressive symptoms have been established by numerous studies (18, 38, 39). Specifically, neuroticism emotionality has been linked to depression, while extraversion and conscientiousness are negatively associated with depressive symptoms (18, 38). A meta-analysis of 10 cohort studies investigating the relationship between personality traits and depressive symptoms found that high levels of neuroticism, low levels of extraversion, and low levels of conscientiousness were all predictive of depressive symptoms (39). Interestingly, this association between personality and depression was strongest in younger age groups (39). Moreover, studies have suggested that personality traits have a genetic basis and have been identified as a mediator of the relationship between depression and heredity (20, 40). For instance, individuals with the short allele of the 5-HTT-linked polymorphic region (5-HTTLPR) exhibit lower expression of the gene product and a higher level of neuroticism than those homozygous (41). In summary, our findings highlight the important role of personality traits in mediating the relationship between depression and heredity through shared genetic factors.

There are several limitations in our study. The sample size used in the analysis was small and limited to a specific population of Chinese college students from only two universities, which may limit the generalizability of the results. To enhance the generalizability of future studies, further studies should include more diverse populations. Additionally, genetic association studies that use only one SNP in the analysis can result in insufficient information about genetic variation. Haplotype analysis, which examines the combined effects of multiple SNPs within a haplotype block, can provide a more comprehensive view of genetic variation and increase the power to detect genetic associations (42). Finally, the study’s cross-sectional design makes establishing causality and directionality difficult (43). Using a longitudinal design in future studies can provide additional information on the relationship between the *SIRT1* rs12415800 polymorphism and depressive symptoms over time.

## 5. Conclusion

These findings are important as they provide valuable insights into the genetic and psychological factors that contribute to the development of depression. By understanding the underlying mechanisms behind this complex condition, researchers may be able to develop personalized interventions that address the unique needs of each individual. For example, targeting personality traits such as conscientiousness and neuroticism may be a useful approach for individuals with the rs12415800 polymorphism who are at risk of developing depression.

## Data availability statement

The raw data supporting the conclusions of this article will be made available by the authors, without undue reservation.

## Ethics statement

The studies involving human participants were reviewed and approved by the Ethics Committee of the Jining Medical University. The patients/participants provided their written informed consent to participate in this study.

## Author contributions

LH guarantor of integrity of entire study. GH conception and design of the study. TY, CL, YX, ZY, and SY the sample collection. FeY, CW, and LJ experimental studies. CW, LJ, DR, FaY, and LL data acquisition. CW, DR, FaY, YB, and ZG literature research. CW and LJ data analysis, interpretation. CW statistical analysis and drafting the manuscript. GH and TY manuscript revision. All authors contributed to the article and approved the submitted version.

## Funding

This project is supported by the National Key Research and Development Program (2022YFE0125300), Innovation Funding in Shanghai (20JC1418600), the National Natural Science Foundation of China (82071262, 81671326, and 19Z103150073), Natural Science Foundation of Shanghai (20ZR1427200, 20511101900), Shanghai Municipal Science and Technology Major Project (2017SHZDZX01), the Shanghai Leading Academic Discipline Project (B205), and Shanghai Jiao Tong University STAR Grant (YG2023ZD26, YG2022ZD024, and YG2022QN111).

## Conflict of interest

The authors declare that the research was conducted in the absence of any commercial or financial relationships that could be construed as a potential conflict of interest.

## Publisher’s note

All claims expressed in this article are solely those of the authors and do not necessarily represent those of their affiliated organizations, or those of the publisher, the editors and the reviewers. Any product that may be evaluated in this article, or claim that may be made by its manufacturer, is not guaranteed or endorsed by the publisher.
