# Supplementary material for: Personality traits as mediators in the association between SIRT1 rs12415800 polymorphism and depressive symptoms among Chinese college students
Source: Front Psychiatry. 2023 Apr 14;14:1104664. doi: 10.3389/fpsyt.2023.1104664 (PMC10146254; doi:10.3389/fpsyt.2023.1104664)
Supplement: Supplementary file 1 [file Table_1.docx]

Supplementary Material

# Supplementary Tables

**Supplementary Table 1** Proportion of college students in each province

| Province | Count | Proportion |
| --- | --- | --- |
| Shandong Province | 594 | 75.48% |
| Guizhou Province | 19 | 2.41% |
| Henan Province | 20 | 2.54% |
| Anhui Province | 10 | 1.27% |
| Heilongjiang Province | 14 | 1.78% |
| Jilin Province | 16 | 2.03% |
| Jiangsu Province | 6 | 0.76% |
| Liaoning Province | 14 | 1.78% |
| Zhejiang Province | 8 | 1.02% |
| Hebei Province | 17 | 2.16% |
| Sichuan Province | 5 | 0.64% |
| Hubei Province | 7 | 0.89% |
| Jiangxi Province | 7 | 0.89% |
| Inner Mongolia Autonomous Region | 2 | 0.25% |
| Yunan Province | 4 | 0.51% |
| Gansu Province | 7 | 0.89% |
| Guangxi Zhuang Autonomous Region | 3 | 0.38% |
| Chongqing Municipality | 11 | 1.40% |
| Shanxi Province | 3 | 0.38% |
| Shaanxi Province | 3 | 0.38% |
| Ningxia Hui Autonomous Region | 2 | 0.25% |
| Qinghai Province | 2 | 0.25% |
| Tianjin Municipality | 2 | 0.25% |
| Guangdong Province | 1 | 0.13% |
| Hunan Province | 10 | 1.27% |
